# Water Relationships of Whey Permeate Powders

**DOI:** 10.1111/1750-3841.70883

**Published:** 2026-01-31

**Authors:** Tsung‐Yueh Benjamin Peng, Didem Sözeri Atik, Job Ubbink

**Affiliations:** ^1^ Department of Food Science and Nutrition University of Minnesota Minneapolis Minnesota USA

**Keywords:** coproducts, crystallization, glass transition, plasticization, powder, upcycling, water activity

## Abstract

Water relationships are developed for whey protein permeate powders based on an analysis of the composition, structural organization, and thermal properties of three commercial whey permeate powders (WPPs). On the basis of microscopic analysis, a two‐phase model of permeate powders with two types of particle morphologies is proposed: (1) particles consisting of small lactose crystallites dispersed in a continuous amorphous phase consisting of lactose and all non‐lactose constituents and (2) particles consisting of one or a few large lactose crystals with a thin, discontinuous amorphous surface layer. The amorphous fraction of the WPPs is characterized by a low glass transition temperature even at very low water activities and is furthermore strongly plasticized by water. The amorphous fraction of permeate powders was determined from the change in heat capacity associated with the glass transition. The fraction of the amorphous phase and its physical properties were found to vary significantly between the three powders and were influenced by both composition and processing. The results of this study provide a deeper understanding of the physical structure, water relations, and thermal properties of WPPs.

**Practical Applications:**

We introduce a novel structural model for whey permeate powders (WPPs) consisting of a crystalline 𝛼‐lactose monohydrate phase and an amorphous phase that is composed of all other permeate constituents as well as some residual lactose. The amorphous phase of the WPPs, which is present at the surface of the powder particles, is characterized by a low glass transition temperature (*T*_g_). The low *T*_g_ explains the instability (caking and browning) of commercial WPPs. Our studies confirm the high sensitivity of WPPs to water and the limited potential to stabilize commercial WPPs by further drying: even when fully dry, which is difficult to attain in industrial drying, the amorphous fraction of WPPs is still in the rubbery state at ambient temperature and WPPs remain thus prone to caking and color formation. Thus, stabilizing the WPPs by drying them to lower water contents is expected to lead to only very limited improvement of the powders’ stability due to the inherently low of the *T*_g_ of the amorphous phase in WPPs. We argue that the way to effectively improve the stability of whey permeate powders is to increase the *T*_g_ of the amorphous fraction powders.

## 1 Introduction

Whey permeates are major coproducts of the dairy industry and result from the ultrafiltration of the whey that results from cheese production (Hargrove et al. 1976; Tamime 2009). Even though relatively low in protein content, whey permeates are nutritionally important as they contain elevated levels of valuable minerals and other micronutrients (Menchik et al. 2019). Currently, most of the permeates are used in liquid form as a feed ingredient (O'Donoghue and Murphy 2023). Whey permeates are also used in powder form, but major issues are encountered with their stability and functionality, impeding the development of high‐added‐value products.

The composition of whey permeates significantly depends on the source of the whey from which it is produced, which may be acid or sweet cheese whey or, less frequently, milk whey, and on the processing of the whey (Hargrove et al. 1976; Tamime 2009; Durham 2009; O'Donoghue and Murphy 2023). Apart from water, lactose is the major constituent of permeates and typically varies from 75% to 85% w/w on dry basis (d.b.) (Smith et al. 2016). Permeates are further categorized as either sweet or acid whey permeates, depending on the type of cheese from which they are produced (Jelen 2004). Acid whey permeate contains much higher levels of acids than sweet whey permeate (∼7% w/w d.b. vs. ∼2% w/w d.b.) (Hargrove et al. 1976). Lactic acid and citric acid are the principal organic acids (Menchik et al. 2019), and potassium, calcium, sodium, magnesium, and phosphorus are the main minerals in permeates (Kalab et al. 1991; Smith et al. 2016).

As an important food and feed ingredient, permeates are often utilized in powder form, produced via concentration of the liquid permeate, crystallization of lactose, spray drying of the concentrated slurry containing lactose crystals, followed by final drying to water content about 4%–5% w/w (w.b.) (Hargrove et al. 1976). For whey permeate powder (WPP) process, once the lactose is crystallized, the slurry is then spray‐dried at a temperature of approximately 150°C. After spray‐drying, the powder generally has a water content of around 10% w/w w.b., which allows for post‐crystallization of lactose to maximize the fraction of crystalline lactose. Final drying, using a fluidized bed, further dehydrates the WPP to a final water content of about 5% w/w w.b.

Several researchers (Hargrove et al. 1976; Kalab et al. 1991; Ibach and Kind 2007; Huppertz and Gazi 2016; Pandalaneni and Amamcharla 2018; Thorakkattu 2020; Sunkesula 2020) have claimed that lactose crystallization is crucial to preventing the WPPs from caking and stickiness due to the high stability of lactose crystals at relative humidity values below about 85% (Kalab et al. 1991). Amorphous lactose is highly sensitive to water, resulting in the caking of whey powder (Saltmarch and Labuza 1980; Roos and Karel 1991; Chuy and Labuza 1994; Ibach and Kind 2007). Numerous studies have focused on lactose crystallization in WPP processing (Tanguy et al. 2017; Pandalaneni and Amamcharla 2018; Sunkesula 2020), crystal formation (Bhargava and Jelen 1996; Wijayasinghe et al. 2020), and ease of spray drying (Hargrove et al. 1976).

Despite the well‐established commercial production of permeate powders, WPPs are observed to be highly sensitive to transport and storage conditions (Goula 2017; Oliveira et al. 2019). Specifically, the physical instability of WPPs causes issues of caking (Carpin et al. 2016) and browning (Thorakkattu 2020). As we argue in this article, although the crystallinity of lactose may be expected to be relatively stable during storage of WPPs, the crystallization of a large fraction of lactose leads to the concentration of the other constituents of whey permeate in an amorphous phase. As these other constituents are often highly polar and of low molecular weight, we may expect this amorphous phase to be sensitive to water and temperature. Thus, it is of critical importance to characterize the physical state of the various phases of WPPs to be able to predict their processing characteristics as well as their stability during storage (Roos and Drusch 2016b; Ubbink 2025).

A critical property in analyzing the caking and sticking of amorphous food powders is the glass transition (Downton et al. 1982; Walstra 2003; Ubbink 2025). Increasing the storage temperature causes amorphous powders to transition from a glassy state to a rubbery state, accompanied by a dramatic increase in molecular mobility (Champion et al. 2000; Schmitz‐Schug et al. 2013; Roos and Drusch 2016a). In the rubbery state, the matrix of the powder particles will start to flow, resulting in the collapse of the particle structure and in the caking of the powder (Fitzpatrick et al. 2007).

Lactose in the crystalline state is much less sensitive to temperature and water than in the amorphous state. For example, at 25°C, the deliquescence point (RH_0_) is reported to be 99% for 𝛼‐lactose monohydrate and 87% for 𝛽‐lactose anhydrous (Allan et al. 2020). Although crystalline lactose will ultimately melt when heated, its melting temperature is very high (200–220°C for 𝛼‐lactose monohydrate) (Gombás et al. 2002; Wijayasinghe et al. 2020). Consequently, crystalline lactose is relatively stable during long‐term storage.

The principal water relationships to be considered in the analysis of the physical stability of WPPs during common storage conditions are the water vapor sorption (WVS) isotherm and the dependence of the glass transition temperature on the water content of the WPPs. The third water relationship that we will employ is the dependence of the glass transition temperature on the water activity. The glass transition is a second order phase transition that, when the temperature of an amorphous material increases to above its *T*_g_, it transitions from a hard and brittle glassy state to a soft and rubbery state (Angell 1988, 2002). Plasticization refers to the reduction of the *T*_g_ of a matrix by incorporating low‐*M*_w_ compounds. Water, with its polar nature and low molecular weight (18 Da), is the most potent plasticizer of amorphous foods (Ubbink 2025).

In light of the two‐phase nature of WPPs, the three water relationships (the WVS isotherm and the dependence of the glass transition temperature on both the water content and the water activity) need to be analyzed in terms of a structural model that includes a crystalline lactose phase and an amorphous phase of complex composition. Common models to fit two of these water relationships are the Brunauer–Emmett–Teller (BET)–Guggenheim–Anderson–de Boer (GAB) equation (Brunauer et al. 1938; Anderson 1946) for the WVS isotherm and the Gordon–Taylor equation (G&T) (Gordon and Taylor 1952) for the dependence of the glass transition temperature on the water content. As applied to food powders, both the BET–GAB and the G&T equations are based on the assumption that the powder matrix consists of a single, amorphous phase. We will furthermore demonstrate that the third water relationship, the dependence of the glass transition temperature on the water activity, can be determined from the first two ones (Ubbink 2025).

To accurately analyze the water relationships of WPPs, we modify the GAB equation to take into account the water that is locked up in the crystalline lactose monohydrate phase of the WPPs. We will demonstrate that the water in this crystalline phase is inert to the change of external water activity for temperatures that are not too high. Similarly, we will propose a modification of G&T equation, based on the insight that a water molecule locked in the crystalline lactose monohydrate phase does not plasticize the amorphous phase of WPPs. We therefore introduce a parameter for the water content of the water locked in the lactose monohydrate crystals for GAB and G&T equation.

The aim of this research is to determine the water relationships of WPPs. On the basis of the analysis of industrial WPPs, we propose a novel model for the structure of WPPs as composite systems comprising crystalline and amorphous phases. Next, the WVS and the dependence of the glass transition temperature of the three WPPs on water content and water activity are analyzed. This will be the first time that these so‐called water relationships are established for WPPs. We expect that the structural model for WPPs proposed in this article will serve as a critical foundation for further research to improve the functionality and storage stability of WPPs.

## 2 Materials and Methods

### 2.1 Permeate Samples and Materials

Pharmaceutical grade 𝛼‐lactose monohydrate was purchased from Kerry Group plc (Tralee, Ireland). Analytical grade petroleum ether, anhydrous diethyl ether, ethyl alcohol, lithium bromide, lithium chloride, potassium acetate, potassium carbonate, magnesium nitrite, sulfuric acid (high‐performance liquid chromatography [HPLC] grade), and 𝛽‐lactose (anhydrous) were purchased from Sigma‐Aldrich Co. (St. Louis, MO, USA) and analytical grade magnesium chloride was purchased from Thermo Fisher Scientific Inc. (Waltham, MA, USA).

Three commercial WPPs were obtained from three different producers in the United States and coded as WP A, WP B, and WP C. The whey permeates were concentrated to 60% to 70% total solids (w/w), followed by lactose crystallization. Crystallization was induced for WP A and WP B by cooling the concentrates from ∼80–90°C to ∼35–40°C at a cooling rate of 5–6°C/min, followed by a holding phase of 2–4 h at the final temperature to maximize lactose crystallization. The process for WP C was similar but for the lactose crystallization. From an initial temperature of ∼75°C, the concentrate was cooled to ∼20°C at a cooling rate of 0.05–0.1°C/min, followed by a holding phase of ∼12 h. After crystallization, the slurry was spray‐dried to a water content of approximately 10% w/w, followed by a final drying in a fluidized bed dryer to a final water content of ∼5% w/w. Fully amorphous versions of WP A, WP B, and WP C were prepared by reconstituting the WPPs under stirring at a total solids of 30% w/w in deionized water at 60°C until the lactose crystals were fully dissolved, followed by spray drying of the dispersion using a B‐190 laboratory spray dryer (Büchi, Flawill, Switzerland).

### 2.2 Compositional Analysis

The lipid content of the permeate powders was determined using the Mojonnier method following AOAC official method 922.06 (AOAC 2006). The lipids in the sample matrix were first hydrolyzed by hydrochloric acid (37% w/w) and then extracted by diethyl ether and petroleum ether using a Mojonnier setup. The lipid content was determined gravimetrically, and the analyses were performed in duplicate.

The total nitrogen content of the permeate powders was determined using a combustion method employing an FP828 nitrogen analyzer (LECO Corp., St. Joseph, MI, USA) following the Dumas method (AOAC official method 990.03) (AOAC 2006). The amount of nonprotein nitrogen (NPN) was determined using the trichloroacetic acid precipitation method (ISO/IDF 2016; DeVries et al. 2017). True protein nitrogen was calculated by subtracting NPN from total nitrogen. A value of 6.38 was adopted for the nitrogen–protein conversion factor, as is recommended for dairy products (AOAC 2006). The NPN content was expressed in terms of protein equivalent. Analyses were performed in duplicate.

The ash content of the permeate powders was analyzed gravimetrically by a dry ashing method according to AOAC official method 954.46 (AOAC 2002). Precisely weighted amounts of samples (∼1 g) were pyrolyzed in a muffle furnace at 650°C, following which the ash amount was determined gravimetrically. Analyses were performed in duplicate.

The lactose content of WPP samples was analyzed by HPLC using 1260 Infinity II chromatograph (Agilent Technologies, Santa Clara, CA) equipped with an Aminex HPX‐87H column (300 mm × 7.8 mm, Bio‐Rad, Richmond, CA). Samples were prepared following Smith et al. (2016). 0.013 N sulfuric acid was used as a mobile phase at 0.6 mL/min flow rate, and samples were injected into the column using an autosampler (G7129A, Agilent Technologies). Analyses were carried out in triplicate.

### 2.3 Mineral Analysis

The content of potassium, calcium, sodium, magnesium, and phosphorous was determined by inductively coupled plasma optical emission spectrometry (ICP‐OES) using facilities at the Research Analytical Laboratory at the University of Minnesota. The samples were prepared by microwave digestion before ICP‐OES analysis (iCap 7600 Duo ICP‐OES Analyzer, Thermo Scientific Inc., Waltham, MA, USA). Analyses were performed in duplicate.

### 2.4 Microscopy

The morphology and structure of the permeate samples were analyzed by direct‐light and polarized‐light microscopy using a BX40 microscope (Olympus Corp., Tokyo, Japan) equipped with polarizing filters and coupled with a digital camera (DP26, Olympus Corp., Tokyo, Japan). Small amounts (∼10 mg) of the sample were mounted on glass slides, fixed with a drop of Canada balsam (Chem‐Impex International, Wood Dale, IL, USA), and covered with a microscope cover glass. The lactose crystal size distribution in samples was measured and calculated using 15–25 micrographs per sample and tracing at least 250 individual crystals.

### 2.5 Water Activity Determination

The water activity of the permeate samples was determined at 25°C ± 1°C using a LabMaster Neo water activity meter (Novasina, Laachen, Switzerland). Samples were analyzed in triplicate.

### 2.6 Water Content Determination and Dehydration Kinetics

The water content (*Q*_w_) of the samples was determined by drying under reduced pressure in a NAPCO Model 58301 laboratory vacuum oven (National Appliance Co., Portland, OR) with a modified set‐up. The air inlet of the vacuum oven was connected to two drying cartridges filled with Drierite (W.A. Hammond Drierite Company, LTD, Xenia, OH) and a pre‐drying cartridge filled with silica gel beads (L2K Commerce Company, Brea, CA) that were set up in series to reduce the moisture in the drying air to trace amounts. Permeate samples were dried at 40°C, 50°C, 55°C, 60°C, 70°C, 80°C, and 100°C under a flow of dehumidified air at a flow rate of 2 L/min and a reduced pressure of ∼20 mbar. The water content of the samples was gravimetrically determined from the weight loss of the samples during drying for several drying times. Analyses were carried out in triplicate. Water contents are reported on wet basis, except for the WVS isotherms, for which the water content is conventionally reported on dry basis.

### 2.7 Water Activity Equilibration

Permeate samples were equilibrated at various water activities by conditioning at *T* = 22°C ± 1°C in a series of desiccators containing saturated salt solutions of known water activity (*a*_w_ = 0.06 (LiBr); *a*_w_ = 0.11 (LiCl); *a*_w_ = 0.22 (CH_3_COOK); *a*_w_ = 0.33 (MgCl_2_); *a*_w_ = 0.43 (K_2_CO_3_); *a*_w_ = 0.54 (Mg(NO_3_)_2_). About 1 g of powder was precisely weighed in a glass weighing dish. The sorption of water was followed gravimetrically, and the equilibrium water content of the samples was calculated from the weight change during equilibration and the initial water content of the samples. The experiments were performed in duplicate.

### 2.8 Differential Scanning Calorimetry (DSC)

A Discovery 2500 DSC (TA Instruments, New Castle, DE) coupled with an RCS 90 infracooler (TA Instruments, New Castle, DE) was used to determine the glass transition of the samples (*T*_g_) and the change of heat capacity associated with the glass transition (Δ*C*_p_). Approximately 10 mg of sample was precisely weighted into a Tzero sample pan and hermetically sealed using a Tzero hermetic lid (TA Instruments, New Castle, DE, USA). The onset glass transition temperature of the samples was determined from the heat flow at the second heating ramp (at 5°C/min) from the intersection of the tangent to the low‐temperature baseline and the tangent at the inflection point. Temperature scanning range was performed from −60°C to +60°C. Examples of a first and second heating ramp are shown in Figure S1. As expected for samples with a low glass transition temperature, there is little enthalpy relaxation observed during the first heating ramp. We nevertheless decided to maintain the two heating ramps to remain consistent between the experiments. The heat capacity Δ*C*_p_ associated with the glass transition was determined as the difference between the heat capacity in the rubbery and glassy states. The analyses were conducted in duplicate.

### 2.9 X‐ray Diffraction (XRD)

The XRD diffractograms for the WPPs (WP A, WP B, and WP C), dehydrated WP A at 60°C and 80°C, 𝛼‐lactose monohydrate, and 𝛽‐anhydrous lactose were determined using a Bruker D8 Discover 2D micro‐diffractometer equipped with a Co‐*k*_α_ point radiation source (Eden Prairie, MN, USA). The detector has 500,000 pixels that cover a range of 30° for the angles 2*θ* and 𝜓. A collimator narrowed the x‐ray beam to a spot diameter *d*_c_ = 0.1 mm on the focused object. The instrument was operated at 40 kV and 35 mA. Samples were measured from 2𝜃 = 5° to 40°. Peaks were analyzed using JADE 9.4. software (MDI), and the angular ranges were converted to Cu‐*k*_a_ radiation. The degree of crystallinity of WPPs was analyzed by integrating the peaks area of the measured sample and subtracting the integration of the amorphous halo using the following equation:

(1) $$F_{C}=\frac{A_{\mathrm{SP}}-A_{\mathrm{AP}}}{A_{\mathrm{SP}}}$$

where *F*_C_ is the crystalline fraction, *A*_SP_ is the integrated area of the XRD pattern of the WPP sample, and *A*_AP_ is integrated area of the XRD pattern of the fully amorphous WPP.

### 2.10 Statistical Analysis and Data Regressions

Statistical analysis was carried out by one‐way ANOVA analysis with confidence levels of *p* < 0.05 using JMP Pro 17 (JMP Statistical Discovery LLC, NC). The Tukey multiple comparison test was used to compare means to evaluate significant differences among samples. Regressions were performed using a least‐squares method. For linear regressions, the quality of fit is determined by the square of the Pearson correlation coefficient for the data sets of the dependent and independent variables. Nonlinear regressions were conducted following steps: First, the square of the residual between the observed value and the predicted value was calculated for each pair of observed and modeled values. Second, the sum of the squares of all residuals was minimized using a generalized reduced gradient algorithm (GRG) as implemented in Excel's Solver tool, using reasonable values to start the regression. Criteria underlying the choice of these starting values are 1. Both starting values and optimized fitting parameters are plausible from a physical point‐of‐view; and 2. The outcome of the minimization converges to the same, stable solution for different starting values. Third, the overall quality of the fit was determined by calculating *R* ^2^ and by confirming that the residuals were randomly scattered.

## 3 Results and Discussion

The composition of the three commercial WPPs, which were all derived from cheese processing, was determined (Table 1). WP B is mainly produced from acid‐coagulated cheeses, whereas WP A and WP C are from the sweet whey from cheddar production; all three samples are, however, from plants in which blended permeate streams are handled. The lactose content of commercial permeate was found to be between 78.0% and 81.5%. Our values correspond well with literature values for WPPs produced from mozzarella and cheddar (Smith et al. 2016). The fat content of samples was found to be low, in line with previous literature (Beucler et al. 2005; O'Donoghue and Murphy 2023). Whey permeate typically contains 2%–7% w/w protein and mostly NPN (ADPI 2022). In the present study, the NPN content (as protein equivalent) of three whey permeate samples was found to be between 2.36% and 3.61%, aligning well with literature results (Frankowski et al. 2014). The ash content of WP B was found to be higher than WP A and WP C (Table 1). Overall, our values are on the lower end of the reported range of 8%–20% (Beucler et al. 2005; Smith et al. 2016). The most abundant mineral in our whey permeate samples is potassium, followed by sodium and calcium (Table 1).

**TABLE 1:** Compositional data of whey permeate powders.

Content	WP A	WP B	WP C
Fat (% w/w d.b.)	1.11 ± 0.12	0.87 ± 0.49	0.23 ± 0.16
True protein (% w/w d.b.)	0.61 ± 0.09	3.62 ± 0.19	3.49 ± 0.50
Nonprotein nitrogen (% w/w d.b.)^a^	2.36 ± 0.12	3.61 ± 0.17	2.92 ± 0.45
Ash (% w/w d.b.)	6.06 ± 0.26	8.57 ± 0.17	6.88 ± 0.12
Lactose (% w/w d.b.)	81.5 ± 4.1	73.7 ± 3.4	77.9 ± 2.8
Water content (% w/w w.b.)^b^	5.83 ± 0.03	5.78 ± 0.00	5.53 ± 0.10
Water activity (–)^b^	0.242 ± 0.004	0.181 ± 0.00	0.204 ± 0.002
Minerals (% w/w dry basis)			
Ca	0.61 ± 0.01	0.89 ± 0.00	0.59 ± 0.00
K	2.78 ± 0.00	2.47 ± 0.00	3.01 ± 0.03
Mg	0.17 ± 0.00	0.18 ± 0.00	0.16 ± 0.00
Na	0.72 ± 0.00	1.31 ± 0.00	0.93 ± 0.02
P	0.74 ± 0.00	0.86 ± 0.00	0.83 ± 0.00

Abbreviations: d.b., dry basis; w.b., wet basis.

^a^ NPN, expressed as protein equivalent.

^b^ As received.

**FIGURE 1:**
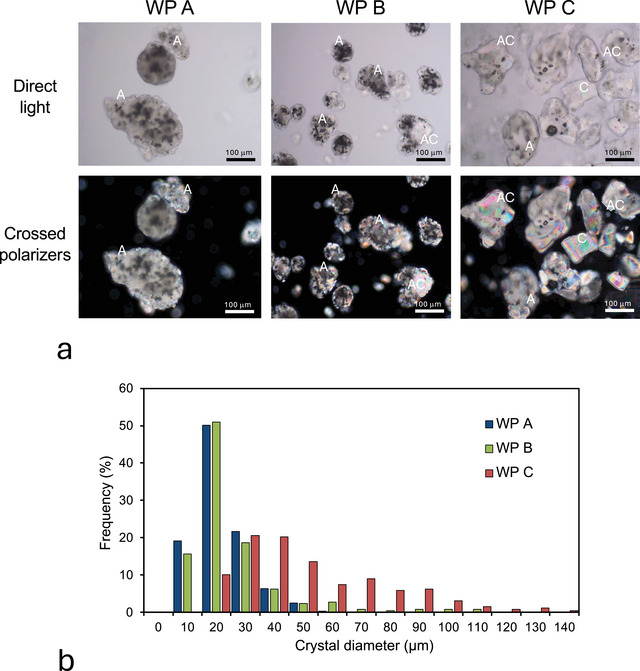
(a) Microscopic images of whey permeate powders WP A, WP B, and WP C. The images were obtained in direct light (top) and under polarized light with cross polarizers (bottom). The scale is indicated in the micrographs. (b) Size distribution of lactose crystals in the three whey permeate powders.

Microscopic images of the three commercial permeate samples were obtained under direct light and with polarized light with crossed polarizers (Figure 1a). From the images, it is clear that all samples contain a crystalline phase. The size and shape of the lactose crystals vary significantly between the samples (Figure 1b). WP A, WP B, and WP C are characterized by mean values of 17.75, 21.06, and 47.00 µm, respectively, and the particle size distributions for WP A and WP B are furthermore very similar. WP C is characterized by a much larger crystal size than samples WP A and WP B, as is in addition witnessed by the large birefringent domains and the regular shapes of these domains (Figure 1a), allowing the identification of the characteristic tomahawk shape of α‐lactose monohydrate (Goff et al. 2022).

From the microscopic images, both with polarized and direct light, we observe that the permeate powder particles have a composite structure with a non‐birefringent amorphous phase next to the lactose crystals. In the case of WP A and WP B, this amorphous material constitutes the continuous phase, fully enveloping the small lactose crystals. Likely because of the ductility of this amorphous phase, the particles of WP A and WP B are significantly rounded. Several of such particles are marked as “A” in Figure 1. In the micrograph of WP C, some amorphous material is apparent, in particular in several of the particles containing smaller lactose crystals, forming particles that are similar to those observed for WP A and WP B. The larger lactose crystals may have little of the amorphous material but likely enough to stick several crystals together (the particles marked as “C” in Figure 1. Mixed‐form particles are also visible for WP C and possibly for WP B, with a larger crystal protruding from the amorphous phase that may also contain several fully embedded smaller crystals [particles marked “AC” in Figure 1]). The smaller lactose crystals observed in WP A and WP B are directly attributed to the crystallization kinetics: a relatively higher cooling rate (5–6°C/min) and shorter holding time (2–4 h). This fast cooling increases the number of nuclei formed (Wong and Hartel 2014), limiting subsequent crystal growth. Conversely, WP C was produced under conditions similar to pure α‐lactose monohydrate, utilizing a slower cooling rate (0.05–0.1°C/min) and a longer holding time (12 h). This slower rate yields fewer nuclei, allowing the crystalline lactose in WP C sufficient time to grow into the classic tomahawk‐shape characteristic of 𝛼‐lactose monohydrate.

**FIGURE 2:**
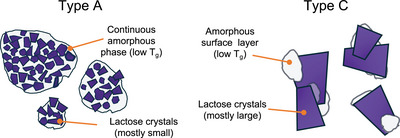
Two‐phase model of permeate powder particles. Type A particles consist of a continuous amorphous phase with small lactose crystals that are largely embedded in the amorphous matrix. Type C particles consist of one or at maximum a few larger lactose crystals that may be glued together by amorphous material and that contain an amorphous layer partially covering the crystal surfaces.

To explain the structure–property relationships of permeate powders, we propose a two‐phase model for WPPs with two principal particle morphologies (Figure 2). Type A particles consist of a continuous amorphous phase that embeds multiple small lactose crystals. The second type of particles, type C, consists of one or, at maximum, a few larger lactose crystals covered by a thin layer of a concentrated amorphous phase, which is composed of noncrystalline (amorphous) lactose, monosaccharides, organic acids, peptides, and minerals. Mixed AC‐type particles, as observed in Figure 1 for WP B, are likely formed by the agglomeration of A‐type and C‐type particles. The particle morphology is determined by the crystallization conditions, with lower cooling rates and reduced level of agitation and surface scraping resulting in larger crystals, such as in WP C.

To develop the water relationships for permeate powders, we need to consider the material properties of the individual, crystalline, and amorphous phases. At ambient temperatures and up to a relative humidity of ∼85%, α‐lactose monohydrate is thermodynamically stable. At higher temperatures, it may release its crystal water. As the permeate powders contain appreciable amounts of polar low molecular weight compounds (Smith et al. 2016; Menchik et al. 2019), we expect the amorphous phase of permeate powders to be highly sensitive to water and likely to be in the rubbery state at room temperature. This is confirmed by the present study. For this reason, we have informally coined the name “gooey phase” for the amorphous fraction, which we define as the weight fraction of the WPPs that is in the amorphous state and which includes the water present in the amorphous phase.

The proposed two‐phase model identifies the amorphous phase of WPPs as the critical factor governing powder instability. This model illustrates that the composition of the amorphous phase changes dynamically due to further lactose crystallization. This process concentrates polar, low‐molecular‐weight constituents—particularly organic acids—into the remaining amorphous phase, leading to strong plasticization that depresses the *T*_g_ below typical storage temperatures (25°C). The resulting rubbery state of the amorphous phase in WPPs is the primary cause of caking and browning during storage and transportation. Compositional analysis revealed a significant amount of lactic acid (∼7% w/w d.b.), as expressed on the weight of amorphous fraction. Given that lactic acid is a strong plasticizer, we hypothesize that it is the primary contributor to the plasticization in the amorphous phase of WPPs. Accordingly, we are conducting a systematic model study to investigate the plasticization of amorphous lactose by lactic acid.

In the permeate particles, water is present in both the crystalline and the amorphous phases. The water in the amorphous phase is mobile, whereby the water vapor pressure, or water activity, controls the amount of water that is capable of being dynamically held by the food matrix (Barbosa‐Cánovas 2020). On the other hand, the water located in the crystalline lactose phase is confined in the crystal lattice and does not show lateral mobility at room temperature. Crystal water is not affected by the (external) vapor pressure of water as long as the relative humidity is lower than the deliquescence point (Allan et al. 2020; Mauer 2022). Therefore, an activation energy is required to break the interaction of water within the lattice structure (Garnier et al. 2008).

We thus expect the water in the amorphous phase of WPP to respond to changes in environmental humidity, whereas the water in the α‐lactose monohydrate phase will remain locked in the crystal lattice, provided that the temperature is not too high. This phase‐dependent behavior of water in permeate powders may be exploited to determine the water content of the amorphous phase as well as the total water content of the powder by drying the powders at different temperatures.

**FIGURE 3:**
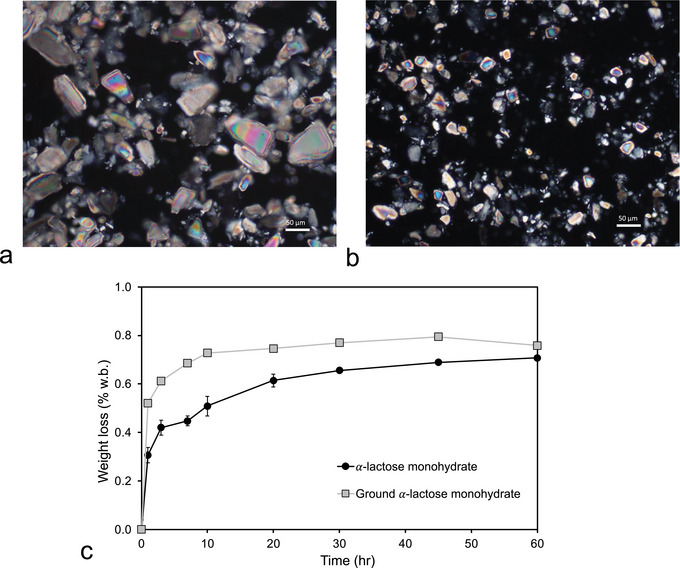
Effect of particle size on lactose drying kinetics. Microscopic images of (a) α‐lactose monohydrate as purchased, and (b) α‐lactose monohydrate ground using mortar and pestle and sieved (sieve size: 60 µm). (c) Dehydration kinetics of the original and milled samples at 60°C for drying times up to 60 h.

**FIGURE 4:**
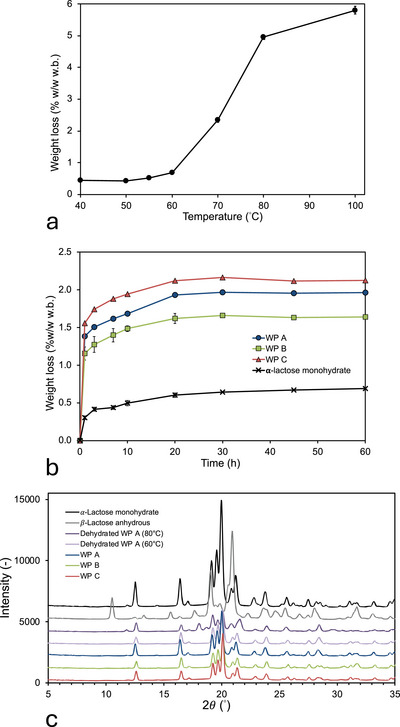
(a) Weight loss of 𝛼‐lactose monohydrate during drying for 60 h at various temperatures between 40°C and 100°C. (b) Dehydration kinetics of the three whey permeate powders and 𝛼‐lactose monohydrate at *T* = 60°C. (c) X‐ray diffractograms of 𝛼‐lactose monohydrate, β‐lactose anhydrous, and whey permeate powders. Diffractograms for WP A are shown for both the original sample and for samples dried for 60 h at 60°C and 80°C.

We first investigate the release of water from 𝛼‐lactose monohydrate (Figure 4a). At low temperatures (*T* < 60°C), only a small, almost constant fraction of water is released from the samples <0.7 % w/w w.b.; no statistically significant differences (*p* < 0.05) are observed for any of the temperatures between 40°C and 60°C (Table S1). It furthermore turns out that, although the kinetics of dehydration are dependent on the particle size, the amount of water extracted converges to the same value for the longer drying times (Figure 3). We furthermore infer that the low amounts of water release at temperatures of 60°C and lower are related to the release of surface water. The amount of water that is released by drying rapidly increases with increasing temperatures above 60°C, reaching 5.8% ± 0.12% w/w w.b. after drying at 100°C for 60 h (Figure 4a). When corrected for the amount of surface water (∼0.5%–0.7% w/w w.b.), the weight loss at a drying temperature of 100°C is close to the water content of α‐lactose monohydrate (5% w/w w.b.). We conclude that, at 100°C, all the crystal water in 𝛼‐lactose monohydrate is released but remains locked in for drying temperatures of 60°C and lower. The temperature dependence of water release from 𝛼‐lactose monohydrate may be exploited to determine the water content of the amorphous phase of the composite permeate powders: By drying at 60°C, water may selectively be removed from the amorphous phase of the permeate powders, leaving the crystal water locked in the 𝛼‐lactose monohydrate while allowing for the extraction of surface water (Figure 4b). From Figure 4b, we establish that full water extraction from the amorphous phase is attained for a drying time of 30 h at 60°C, as no statistically significant differences (*p* < 0.05) are observed for longer drying times (Table S2). The total amount of water in the WPPs is then determined by drying for 30 h at 100°C, and the amount of water in the 𝛼‐lactose monohydrate phase is determined by difference (Table 2).

To study the water relationship of WPPs, several water content definitions need to be employed: (1) total water content, denoted by *Q*_w_ and expressed on wet basis, measured at 100°C drying; (2) water content associated with the amorphous phase *Q*_w,A_ (w.b.), measured at 60°C drying; and (3) water content associated with the crystalline phase *Q*_w,C_ (w.b.), calculated using Equation (2). In line with the convention for WVS isotherms (Bell and Labuza 2000), the various water contents also need to be expressed on dry basis (d.b.); we denote this by a prime, that is, *Q*′_w_ (d.b.) versus *Q*_w_ (wet basis).

(2) $$Q_{w}=Q_{w,C}+Q_{w,A}$$

**TABLE 2:** Water content of permeate powders as determined by drying at 60°C and 100°C.

Water content basis	WP A	WP B	WP C
*Q*_w_ (% w/w w.b.)	5.69 ± 0.10	5.78 ± 0.03	5.53 ± 0.01
*Q*_w,A_ (% w/w w.b.)	1.63 ± 0.06	1.75 ± 0.19	2.03 ± 0.03
*Q*_w,C_ (% w/w w.b.)	4.06 ± 0.04	4.03 ± 0.11	3.50 ± 0.06

*Note*: Q_w_ is determined by drying for 30 h at 100°C; *Q*_w,A_ is determined by drying for 30 h at 60°C; *Q*_w,C_ is determined by difference.

XRD analysis was performed for the three WPPs and for WP A dehydrated at several temperatures to assess potential transformation of the crystalline organization during dehydration (Figure 4c). To interpret lactose polymorphs, XRD was in addition performed on *α*‐lactose monohydrate and 𝛽‐lactose (anhydrous). Figure 4c allows to determine the temperature dependent transition of the crystal polymorph in the dried WPPs: at a drying temperature of 60°C, the diffractogram of WP A confirms that the crystalline lactose in this sample remains in the α‐monohydrate polymorph, whereas at a drying temperature of 80°C a mixed crystalline phase with a dominant contribution of 𝛼‐lactose monohydrate and a smaller contribution of α‐lactose (anhydrous) is observed, with characteristic values of 2𝜃 at 18.7° and 21.6°C and small peaks at 29.5°, 29.7°, and 30.4° (Garnier et al. 2002; MacFhionnghaile et al. 2017). 𝛽‐lactose (anhydrous) is conversely not observed for any of the WPPs. The XRD experiments thus confirm that the monohydrate form is preserved when the WPPs are dried for 60 h at 60°C.

**FIGURE 5:**
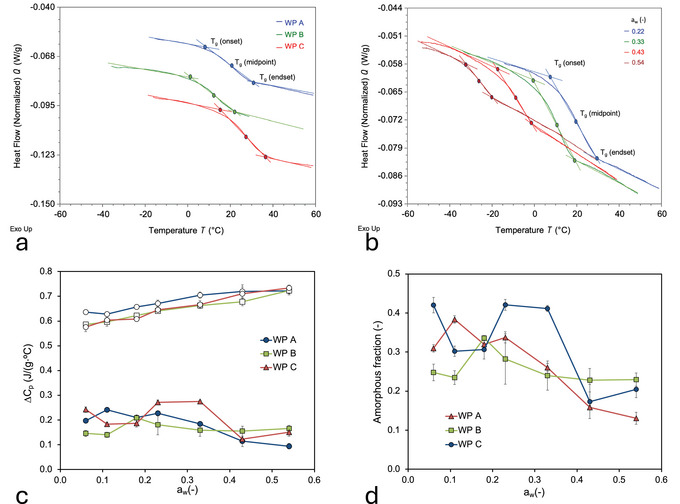
(a) Thermograms of whey permeate powder samples for WP A, WP B, and WP C equilibrated at *a*_w_ = 0.22 (*T* = 22°C ± 1°C). (b) Thermograms of WP A equilibrated at several water activities and at *T* = 22°C ± 1°C, as indicated in the graph. (c) Dependence of the change in heat capacity ΔC_p_ at the glass transition on the water activity for the original whey permeate powder samples (filled symbols) and for fully amorphous whey permeate powders following reconstitution and spray‐drying (open symbols) (d) fraction of amorphous phase as calculated using Equation (3) from ΔC_p_ of the original and fully amorphous whey permeate powders. The thermograms shown in (a) and (b) are the 2nd heating ramp. *T*_g_ (onset), *T*_g_ (midpoint), and *T*_g_ (endset) are indicated on the thermograms in (a) and (b), whereby *T*_g_ (onset) is reported throughout the manuscript as the glass transition temperature.

As expected for materials containing an amorphous phase, the three permeate powders show a glass transition in the form of a smooth, sigmoidal transition (Figure 5a). Given the low glass transition temperatures, between 10°C and 20°C at *a*_w_ = 0.23, the amorphous phase is in the rubbery state at room temperature (25°C), with important consequences for the physical and chemical stability of the whey permeate powders.

The change in heat capacity was previously used to determine the amorphous content of a matrix (Hancock and Zografi 1997; Lehto et al. 2006) based on the assumption that the change in heat capacity over the glass transition Δ*C*_p_ is proportional to the fraction of the amorphous phase (Lehto et al. 2006). Δ*C*_p_ determinations are found to provide consistent results and are generally in good agreement with other techniques, except for samples with a very small Δ*C*_p_ (Lehto et al. 2006). We adopted this method to determine the amorphous fraction of the permeate powders. For this purpose, Δ*C*_p_ values were determined for both the original WPPs (Figure 5c; Table S3) and fully amorphous permeate powders that were prepared by reconstituting and spray drying WP A, WP B, and WP C (Figure 5c; Table S3). The amorphous fraction of the whey permeate samples was then calculated as the ratio of Δ*C*_p_ of the original WPP and the corresponding fully amorphous form (Figure 5d):

(3) $$F_{A}=\frac{\Delta C_{P,P}}{\Delta C_{P,A}}$$

where *F*_A_ is the amorphous fraction; Δ*C*_P,P_ is the heat capacity change associated with the glass transition of a permeate sample, and Δ*C*_P,A_ is the heat capacity change associated with the glass transition of the corresponding fully amorphous sample.

To confirm the Δ*C*_p_ method, the degree of crystallinity of the initial WP A, WP B, and WP C samples was also determined using XRD (Table 3). Overall, we find a good agreement between the two techniques, as the crystalline and amorphous fractions as determined by the two techniques add up to close to 100% (Table 3). We observe that the amorphous fraction of the WPPs is significantly smaller than the crystalline fraction (Figure 5d), in line with the high degree of crystallinity of the lactose. Furthermore, we observed that, with significant fluctuation, the amorphous fraction trends downwards with increasing water activity.

**TABLE 3:** Crystalline fraction of the whey permeate powders as determined by x‐ray diffraction (XRD) and amorphous fraction as determined by differential scanning calorimetry (DSC).

	WP A	WP B	WP C
Crystalline fraction^a^	0.74 ± 0.02	0.69 ± 0.01	0.64 ± 0.02
Amorphous fraction^b^	0.32 ± 0.01	0.29 ± 0.01	0.39 ± 0.02

^a^ From XRD.

^b^ From DSC.

To fit the WVS of the WPPs, we start with the GAB equation (Anderson 1946):

(4)

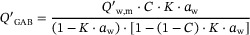

where *Q*′_GAB_ is the water content on d.b. of the amorphous powder, *Q*′_w,m_ is the parameter that is commonly deferred to as the “monolayer water content,” and *C* and *K* are fitting parameters. For *K* = 1, the BET equation is obtained (Brunauer et al. 1938).

As discussed in the Introduction, we modified Equation (4) for the presence of the stoichiometric amount of water that is locked up in the crystalline phase of the WPPs. We thus analyze the WVS of the WPP as a sum of the water in the crystalline and amorphous phases:

(5)

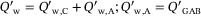

where we assume that the WVS of the amorphous phase can be described by the GAB equation (Equation 4) and that the water content of the crystalline lactose phase remains constant at all water activities during the water vapor equilibration.

**FIGURE 6:**
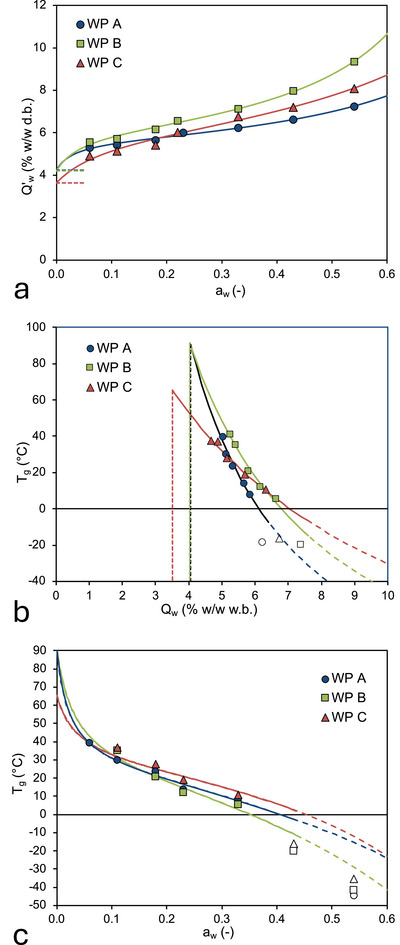
Water relationships for the whey permeate samples, expressed on basis of the total water content of the samples. (a) Water vapor sorption isotherms at *T* = 22°C. The solid lines are the optimal fit to the experimental data using the GAB equation modified to take into account the water contained in the crystalline phase (Equations 4 and 5). (b) Dependence of the glass transition temperature on the water content on wet basis, corrected for the initial water content of the α‐lactose monohydrate crystals. The solid line is the fit of the experimental data with the Gordon–Taylor equation (Equation 6). (c) Dependence of the glass transition temperature on the water activity. The solid lines are derived from a combination of the modified GAB equation (Equations 4 and 5) and the Gordon and Taylor equation (Equation 6), with values of the fitting coefficients as determined for the water vapor sorption isotherms and the water content dependence of the glass transition temperature. The symbols in (**a**), (b), and (**c**) are the experimental data for WP A, WP B, and WP C as indicated in the graphs; error bars are smaller than the symbols. Open symbols are for data not taken into account in the fit. The short‐dashed lines in (**a**) and (b) indicate the water present in the α‐lactose monohydrate crystals at the start of water vapor equilibration. The long‐dashed lines in (c) indicate the extrapolation of the model values to both low and high water activities. Error bars, denoting ± 1 standard deviation, are smaller than the symbols.

The WVS isotherms of the WPPs are fitted using Equations (4) and (5), with the fitting coefficients collected in Table 4. The WVS isotherms of the WPPs show the characteristic sigmoidal shape that is commonly observed for amorphous food systems (Bell and Labuza 2000; Carter and Schmidt 2012), but with a vertical offset that corresponds to the water in the monohydrate crystals (Figure 6a). The impact of the crystalline phase on the WVS isotherms of the permeate powders is clearly visible in Figure 6a: For *a*_w_ = ∼0.1 to ∼0.4, the water content of the samples is much higher than what is observed for fully amorphous dairy powders, such as skim milk powder. For instance, at *a*_w_ = 0.11, the water content of the three permeate powders varies between 5.1% w/w and 5.7% w/w, which is much higher than the water content of 2.3% observed for skim milk powder at this water activity (Vuataz 2002). The water content of the samples furthermore extrapolates to *Q*′_w,C_ for *a*_w_ = 0, rather than to zero as for fully amorphous powders (Figure 6a).

**TABLE 4:** Fitting parameters for the modified Guggenheim–Anderson–de Boer (GAB) equation (Equations 4 and 5) and the Gordon–Taylor equation (Equation 6) for the whey permeate samples.

Parameter	WP A	WP B	WP C
*Q*′_w,C_ (% w/w d.b.)	3.92	4.03	3.50
*Q*′_w,m_ (% w/w d.b.)	1.82	1.86	1.79
*C* (–)	36.7	39.3	35.1
*K* (–)	0.85	1.21	1.22
*R* ^2^	0.989	0.995	0.981
*T*_g,m_ (°C)	90.4	91.1	65.4
*k*_GT_ (–)	31.9	23.8	13.6
*R* ^2^	0.992	0.991	0.992

*Note*: The water content dependence of the water vapor sorption and the glass transition temperature are expressed on the total water content on dry basis (*Q*′_w_) and wet basis (*Q*_w_) of the whey permeate samples, respectively.

The dependence of *T*_g_ on the water content is modeled by the G&T equation (Gordon and Taylor 1952):

(6) $$T_{g}\left(Q_{w,A}\right)=\frac{T_{g,m\;}\cdot \left(1-Q_{w,A}\right)+k_{\mathrm{GT}}\cdot Q_{w,A\;}\cdot T_{g,w}}{\left(1-Q_{w,A}\right)+k_{\mathrm{GT}\;}\cdot Q_{w,A}}$$

where *Q*_w,A_ = *Q*_w_ − Q_w,C_ is the content of water associated with the amorphous phase of the WPPs, expressed on wet basis, *T*_g,m_ is the glass transition temperature of the anhydrous amorphous phase, *T*_g,w_ is the glass transition temperature of water (−135°C), and *k*_GT_ is the G&T parameter.

The glass transition temperature of the WPPs decreases with increasing water content (Figure 6b). The plots in Figure 6b are expressed on the total water content of the permeate powders and are thus horizontally offset by the water content of the crystalline phase: The water of the crystalline phase is constant and defined by the crystal stoichiometry. The amorphous phase, however, is strongly plasticized by water, as is common for carbohydrate‐based matrices (Roos and Karel 1991; Chuy and Labuza 1994; Ubbink et al. 2008), and even small changes in water content can transition the amorphous matrix of foods from a brittle, stable, glassy state to a ductile, high‐mobility rubbery state. In the rubbery state, food powders may stick together and agglomerate, losing their flowability, eventually followed by a full structural collapse (Vuataz 2002; Ubbink 2012; Roos and Drusch 2016a, 2016b; Carpin et al. 2016). At higher water contents, the measured glass transition temperature of the whey permeate samples deviates significantly from the modeled values (open symbols in Figure 6b). As we will argue below, this is related to the further crystallization of lactose in the high‐mobility states at these higher water contents, an effect that is not modeled in Equation (6).

Relevant for the storage stability of dairy powders is the water activity rather than the water content (Saltmarch and Labuza 1980; Chuy and Labuza 1994; Vuataz 2002). Figure 6c shows the dependence of the glass transition temperature of the WPPs on the water activity. The solid lines in Figure 6c were generated by combining the fit of the WVS data by the GAB equation (Equations 4 and 5) and the glass transition data by the G&T equation (Equation 6), with fitting coefficients as determined independently for each equation (Table 4). Again, the dashed lines indicate the regime in which we expect further lactose crystallization to take place. Glass transition representations as in Figure 6c are therefore highly useful in analyzing the stability of amorphous powders with respect to variations in water activity and may immediately be used to determine stability regime. For the three WPPs, it is obvious that the stability regimes are very narrow. The critical water activity of the WPPs for which the *T*_g_ equals a storage temperature of 25°C is *a*_w_^*^ = 0.124, 0.134, and 0.176 for WP A, WP B, and WP C, respectively. These water activities will, however, be too high to ensure storage stability under practical conditions. For a more realistic minimum glass transition temperature of 40°C, *a*_w_^*^ to 0.049, 0.063, and 0.053 for WP A, WP B, and WP C, respectively. As the water activities of the WPPs as received were 0.24, 0.18, and 0.20, our results unequivocally demonstrate that the WPPs are in the rubbery state under typical transport and storage conditions (Table 1).

We can further analyze water relationships of the WPPs by expressing the GAB and G&T equations on basis of the water content of the amorphous phase on d.b. (*q*′_w,A_) and wet basis (*q*_w,A_), respectively. The water contents of the amorphous and crystalline phases can be calculated from *Q*_w,A_ and *Q*_w,C_ by dividing the water contents associated with each phase by the weight fractions of the amorphous phase (*F*_A_) and crystalline phase (*F*_C_):

(7a) $$q_{w,A}=Q_{w,A}/F_{A}$$

(7b) $$q_{w,C}=Q_{w,C}/F_{C}$$

where the weight fraction of the amorphous phase can be determined from the change in heat capacity associated with the glass transition (Figure 5c,d). Although we note that there appears to be some fluctuation in Δ*C*_P,P_ in Figure 4c, the amorphous fraction indeed appears to decrease towards higher water activities (*a*_w_ = 0.33, 0.43, and 0.54) (Figure 5d), signifying continuing crystallization of lactose during water activity equilibration in the rubbery state.

**FIGURE 7:**
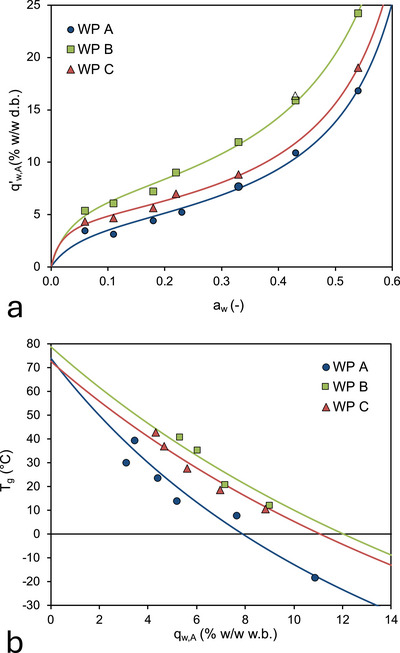
Water relationships for the whey permeate samples, expressed on basis of the water content of the amorphous phase of the samples. (a) Water vapor sorption isotherms at *T* = 22°C. The solid lines are the optimal fit to the experimental data using the GAB (Equation 8). (b) Dependence of the glass transition temperature on the water content on wet basis. The solid line is the fit of the experimental data with the Gordon–Taylor equation (Equation 9). The symbols in (a) and (b) are the experimental data for WP A, WP B, and WP C as indicated in the graphs; error bars, denoting ± 1 standard deviation, are smaller than the symbols.

Normalizing the WVS isotherms (Figure 7a) and the dependence of the glass transition temperature on the weight fraction of amorphous phase (Figure 7b) allows for a direct comparison with the WVS and glass transition of dairy powders (Jouppila et al. 1997; Vuataz 2002). We rewrite the GAB and G&T equations in terms of the water content of the amorphous phase:

(8)

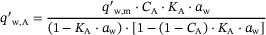

(9) $$T_{g}\left(q_{w,A}\right)=\frac{T_{g,m,A\;}\cdot \left(1-q_{w,A}\right)+k_{\mathrm{GT},A}\cdot q_{w,A\;}\cdot T_{g,w}}{\left(1-q_{w,A}\right)+k_{\mathrm{GT},A\;}\cdot q_{w,A}}$$

where *C*_A_, *K*_A_, *T*_g,m,A_, and *k*_GT_ are the fitting parameters expressed on basis of *q*′_w,A_ and *q*_w,A_.

From Figure 7b, we observe that the *T*_g_ of all WPPs strongly decreases with increasing water content. The experimental data are well fit by the G&T equation, provided that the different water content bases are taken into account; the fitting coefficients are collected in Table 5. As expected, the glass transition temperatures of the fully anhydrous WPPs are significantly lower than of milk powder (Vuataz 2002; Shrestha et al. 2008).

**TABLE 5:** Fitting parameters for the Guggenheim–Anderson–de Boer (GAB) equation (Equation 8) and the Gordon–Taylor equation (Equation 9) for the whey permeate samples.

Parameter	WP A	WP B	WP C
***q*′_w,m_ (% w/w d.b.)**	4.47	6.93	4.91
***C* (–)**	12.9	21.1	34.8
***K* (–)**	1.38	1.33	1.38
***R* ^2^**	0.989	0.996	0.997
***T*_g,m,A_ (°C)**	74.0	78.9	72.8
***k*_GT_ (–)**	6.39	4.26	4.3
***R* ^2^**	0.979	0.981	0.983

*Note*: The water content dependence of the water vapor sorption and the glass transition temperature are expressed on the water content of the amorphous phase on dry basis (*q*′_w,A_) and wet basis (*q*_w,A_) of the whey permeate samples, respectively.

In the present article, we have proposed a structural model for WPPs and have analyzed their water relationships in terms of the WVS and the dependence of the glass transition temperature on the water content and water activity. On the basis of the proposed composite model for WPPs, we are able to quantify the fractions of the amorphous and crystalline phases, finding overall good agreement with the stoichiometry of water in the monohydrate crystals and confirming the high sensitivity of WPPs to humidity and temperature. We are currently investigating the relation between the composition of the amorphous fraction of the permeate powders, which controls the *T*_g_ of the permeate powders, with low‐molecular‐weight compounds generally having lower *T*_g_’s than high‐molecular‐weight compounds (Roos and Karel 1991), and stability of the powders during storage.

Understanding the water relations of WPPs is essential for optimizing drying conditions in industrial manufacturing and to control WPP stability during transport and storage. This approach determines the critical limits of water content and water activity (*a*_w_) required to prevent caking and browning. Our results serve as a guideline for the industry to control temperature and environmental humidity during storage and transportation, ensuring conditions remain as close as possible to the stable region (the glassy state of the powders). Furthermore, our two‐phase model provides the first systematic basis to assess the structural organization of WPPs as a function of composition and processing conditions and allows to rationalize the low glass transition temperature of the amorphous phase. This, in turn, explains high propensity of WPPs to caking and browning. Thus, this provides a direction for the dairy industry to improve product stability: Increasing the *T*_g_ of the amorphous phase can be viewed as a practical strategy to mitigate caking and browning. Potential methods to achieve this are currently under investigation in our research group.

## 4 Conclusions

To develop the water relationships for WPPs, we have investigated the composition, structural organization, and thermal properties of three commercial WPPs. We propose a novel two‐phase model of permeate powders with two types of particle morphologies: (1) particles consisting of small lactose crystallites dispersed in a continuous amorphous phase consisting of lactose and all non‐lactose constituents and (2) particles consisting of one or a few large lactose crystals with a thin, discontinuous amorphous surface layer. Our findings show that the amorphous fraction of the WPPs is characterized by a low glass transition temperature even at very low water activities and is furthermore highly susceptible to plasticization by water. The fraction and physical properties of the amorphous phase were found to vary significantly between the three powders and were influenced by both composition and processing. Overall, this study critically contributes to a deeper understanding of the physical structure, water relations, and thermal properties of WPPs and sets a stage for future research on the mechanism of caking of permeate powders during transport and storage and chemical reactions, in particular the Maillard reaction, that may take place in the amorphous phase.

## Author Contributions

**Tsung–Yueh Benjamin Peng**: Investigation; Writing – original draft; Visualization; Writing – review & editing; Data curation; Validation; Formal analysis.**Didem Sözeri Atik**: Investigation; Data curation; Formal analysis; Writing – review & editing; Validation. **Job Ubbink**: Conceptualization; Investigation; Funding acquisition; Writing – original draft; Methodology; Validation; Visualization; Writing – review & editing; Formal analysis; Project administration; Supervision.

## Conflicts of Interest

The authors declare no conflicts of interest.

## Supporting information




**Supplementary Material**: jfds70883‐sup‐0001‐SuppMat.docx
