# Supplementary material for: Water Relationships of Whey Permeate Powders
Source: J Food Sci. 2026 Jan 31;91(2):e70883. doi: 10.1111/1750-3841.70883 (PMC12859738; doi:10.1111/1750-3841.70883)
Supplement: Supplementary file 1 — Supplementary Material: jfds70883‐sup‐0001‐SuppMat.docx [file JFDS-91-0-s001.docx]

Supplementary Material

Manuscript title: Water relationships of whey permeate powders

Authors: T.-Y.B. Peng, D. Sözeri Atik and J. Ubbink

**Table S1** Water content following dehydration for 60 h at varying temperatures. Reported are mean values ± one standard deviation (n = 3). Superscript lowercase letters indicate statistically significant differences (p < 0.01).

|  | Q_w_ (% w/w w.b.) | | | | | | |
| --- | --- | --- | --- | --- | --- | --- | --- |
| T (ºC) | 40 | 50 | 55 | 60 | 70 | 80 | 100 |
| 𝛼-lactose monohydrate | 0.44 ± 0.03^a^ | 0.43 ± 0.03^a^ | 0.52 ± 0.01^a^ | 0.69 ±0.03^ab^ | 2.34 ± 0.03^c^ | 4.95 ± 0.03^d^ | 5.80 ± 1.20^e^ |

**Table S2** Water content following dehydration at 60°C for varying drying times. Reported are mean values ± one standard deviation (n = 3). Superscript lowercase letters indicate statistically significant differences (p < 0.05).

|  | Q_w_ (% w/w w.b.) | | | | | | | | |
| --- | --- | --- | --- | --- | --- | --- | --- | --- | --- |
| Time (h) | 0 | 1 | 3 | 7 | 10 | 20 | 30 | 45 | 60 |
| Lactose | 0.00 ± 0.00^a^ | 0.30 ± 0.03^b^ | 0.42 ± 0.02^c^ | 0.44 ± 0.03^c^ | 0.50 ± 0.02^d^ | 0.60 ± 0.04^e^ | 0.64 ± 0.03^f^ | 0.67 ± 0.03^f^ | 0.69 ± 0.03^f^ |
| WP A | 0.00 ± 0.00^a^ | 1.38 ± 0.10^b^ | 1.51 ± 0.10^bc^ | 1.62 ± 0.11^c^ | 1.68 ± 0.11^c^ | 1.93 ± 0.09^d^ | 1.97 ± 0.10^d^ | 1.95 ± 0.11^d^ | 1.96 ± 0.09^d^ |
| WP B | 0.00 ± 0.00^a^ | 1.16 ± 0.08^b^ | 1.28 ± 0.09^bc^ | 1.40 ± 0.10^cd^ | 1.48 ± 0.09^d^ | 1.62 ± 0.04^e^ | 1.66 ± 0.07^e^ | 1.63 ± 0.08^e^ | 1.64 ± 0.07^e^ |
| WP C | 0.00 ± 0.00^a^ | 1.56 ± 0.05^b^ | 1.74 ± 0.05^c^ | 1.88 ± 0.04^d^ | 1.94 ± 0.04^d^ | 2.12 ± 0.05^e^ | 2.16 ± 0.09^e^ | 2.12 ± 0.06^e^ | 2.13 ± 0.08^e^ |

**Table S3** Amorphous fraction of the whey permeate samples following water activity equilibration as determined from ΔCp measurements. Reported are mean values ± one standard deviation (n = 3). Superscript and upper and lowercase letters indicate statistically significant differences (p < 0.05).

| a_w_ (-) | F_A_ (%) | | | | | | |
| --- | --- | --- | --- | --- | --- | --- | --- |
|  | 0.06 | 0.11 | 0.18 | 0.23 | 0.33 | 0.43 | 0.54 |
| WP A | 31 ± 1^Ac^ | 38 ± 1^Aa^ | 32 ± 1^ABc^ | 34 ± 1^ABb^ | 26 ± 1^Ad^ | 16 ± 1^Ae^ | 13 ± 2^Af^ |
| WP B | 25 ± 2^Bc^ | 23 ± 2^Bc^ | 34 ± 1^Ba^ | 28 ± 6^Aab^ | 24 ± 4^Ac^ | 23 ± 3^Bc^ | 20 ± 2^Bc^ |
| WP C | 42 ± 2^Ca^ | 30 ± 1^Cb^ | 31 ± 2^Ab^ | 42 ± 1^Ba^ | 41 ± 1^Ba^ | 17 ± 4^ABc^ | 20 ± 2^Bc^ |

**Figure S1** First and second heating ramps of WP B equilibrated at a_w_ = 0.18.
